# Acknowledgement of manuscript reviewers 2015

**DOI:** 10.1186/s13002-016-0084-0

**Published:** 2016-02-16

**Authors:** Andrea Pieroni

**Affiliations:** University of Gastronomic Sciences, Piazza Vittorio Emanuele 9, 12060 Bra/Pollenzo, Cueno, Italy

## Abstract

The editors of *Journal of Ethnobiology and Ethnomedicine* would like to thank all our reviewers who have contributed to the journal in Volume 11 (2015).

**Alain Cuerrier**

Canada

**Alan Louey Yen**

Australia

**Alejandro Casas**

Mexico

**Alfred Maroyi**

South Africa

**Alpina Begossi**

Brazil

**Ana Haydee Ladio**

Argentina

**Ana Maria Carvalho**

Portugal

**Andrea Pieroni**

Italy

**Anely Nedelcheva**

Bulgaria

**Anna Waldstein**

United Kingdom

**Anthony Cavender**

United States of America

**Arnold van Huis**

Netherlands

**Arshad Abbasi**

Pakistan

**Ben Reade**

Italy

**Benno Meyer-Rochow**

Finland

**Benno Meyer-Rochow**

United States of America

**Caroline Weckerle**

Switzerland

**Cassandra Quave**

United States of America

**Christine Sophie Kabuye**

Uganda

**Christoph Schunko**

Austria

**Chunlin Long**

China

**David Casagrande**

United States of America

**E. N. Anderson**

United States of America

**Erdem Yesilada**

Turkey

**Ernani Lins neto**

Brazil

**Fabio Dei**

Italy

**Fusun Ertug**

Turkey

**Gisella S. Cruz-Garcia**

United States of America

**Grace Njoroge**

Kenya

**Haile Yineger**

Ethiopia

**Harriet Kuhnlein**

Canada

**Heike Vibrans**

Mexico

**Hugo De Boer**

Norway

**Ina Vandebroek**

United States of America

**Ingvar Svanberg**

Sweden

**Javier Tardio**

Spain

**Joan Vallès**

Spain

**John Richard Stepp**

United States of America

**Justin Nolan**

United States of America

**Kamal Misra**

India

**Krishna Shrestha**

Nepal

**Lisa Leimar Price**

Netherlands

**Lisa Leimar Price**

United States of America

**Lukasz Luczaj**

Poland

**Manuel J. Macia**

Spain

**Manuel Pardo-de-Santayana**

Spain

**Marco Leonti**

Italy

**Maria Adele Signorini**

Italy

**Maria Lelia Pochettino**

Argentina

**Melissa Ceuterick**

Belgium

**Mirutse Giday**

Ethiopia

**Monika Kujawska**

Poland

**Natalia Hanazaki**

Brazil

**Nemer Narchi**

Mexico

**Nicolas Lescureux**

France

**Nicolas Lescureux**

Norway

**Norma Hilgert**

Argentina

**Peter Giovannini**

United Kingdom

**Phurpa Wangchuk**

Bhutan

**Piero Bruschi**

Italy

**Rainer W Bussmann**

United States of America

**Raziq Abdul**

Pakistan

**Recep Karadag**

Turkey

**Renata Soukand**

Estonia

**Roberta Carvalho**

Brazil

**Romulo Alves**

Brazil

**Ruth Kutalek**

Austria

**Sabine Nebel**

Switzerland

**Sabine Nebel**

United Kingdom

**Sara Vitalini**

Italy

**Sarah Edwards**

United Kingdom

**Shujaul Mulk Khan**

Pakistan

**Sophie Caillon**

France

**Steven Newmaster**

Canada

**Subramanyam Ragupathy**

Canada

**Tinde van Andel**

Netherlands

**Ugur Çakilcioglu**

Turkey

**Ulysses Albuquerque**

Brazil

**Valentina Savo**

Canada

**Valeria Kolosova**

Russian Federation

**Vania Smith-Oka**

United States of America

**Veerle Van Den Eynden**

United Kingdom

**Victor Benno Meyer-Rochow**

Japan

**Victoria Reyes-Garcia**

Spain

**Vivienne Williams**

South Africa

**Wedson Medeiros Souto**

Brazil

**Yadav Uprety**

Canada

**Yunus Dogan**

Turkey

**Zora Dajic Stevanovic**

Serbia

**Zsolt Molnár**

Hungary

